# Genetic variability of E6 and E7 genes of human papillomavirus type 58 in Jingzhou, Hubei Province of central China

**DOI:** 10.1186/s12985-022-01801-6

**Published:** 2022-04-22

**Authors:** Zhiping Yang, Chunlin Zhang, Ping Luo, Mengxia Ye, Quan Gong, Bing Mei

**Affiliations:** 1 Department of Laboratory Medicine, Jingzhou Hospital, Yangtze University, Jingzhou, 434020 China; 2 Department of Immunology, School of Medicine, Yangtze University, Jingzhou, China

**Keywords:** Human papillomavirus type 58 (HPV-58), Variants, Epitope, Phylogenetic analysis, Selection pressure

## Abstract

**Background:**

Cervical cancer is a common malignant tumor in women, with a high mortality rate, has great harm to women’s health. Long-term and persistent infection of high-risk human papillomavirus (HR-HPV) is the main reason of the occurrence and development of cervical cancer.

**Methods:**

The infection rate of HPV-58 is higher in the Jingzhou area. In this study, 172 complete HPV-58 E6-E7 sequences were amplified by polymerase chain reaction (PCR), the amplified products were sequenced, and the gene variations of HPV-58 E6-E7 were analyzed. A Neighbor-Joining phylogenetic tree was constructed by MEGA 11. The secondary structure of E6 and E7 protein was investigated. PAML X was used to analyze the selective pressure. The B cell epitopes of E6 and E7 proteins in HPV-58 were predicted by ABCpred server.

**Results:**

In E6 sequences, 10 single nucleotide variants were observed, including 7 synonymous and 3 non-synonymous variants. In E7 sequences, 12 single nucleotide variants were found, including 3 synonymous variants and 9 non-synonymous variants. There are 5 novel variants. The phylogenetic analysis showed that all the E6-E7 sequences were distributed in A lineage. No positively selected site was found in E6 sequence, but G63 in E7 sequences was identified as positively selected site. Some amino acid substitutions affected multiple B cell epitopes.

**Conclusion:**

Various E6 and E7 mutational data may prove useful for development of better diagnostic and vaccines for the region of Jingzhou, Hubei province of central China.

## Background

Cervical cancer is a common female malignant tumor. According to the statistic results in 2020 all over the world [1], cervical cancer is the fourth most frequently diagnosed cancer and fourth leading cause of cancer death in women, with an estimated 604,172 new cases and 341,831 deaths worldwide in 2020. That account for 6.5% and 7.7% of new cancer cases and deaths worldwide, respectively. The burden of cervical cancer is higher in developing country, has a tremendous impact on the lifetime of millions of women [2].

Human papillomavirus (HPV) is a spherical and non-enveloped virus with a double-stranded circular DNA genome, showing strong squamous epithelium-like characteristics to infect damaged epithelial cells [3]. More than 200 types of HPV have been fully characterized [4]. HPVs have been divided into low-risk types (HPV-6,11, ect.) and high-risk types (HPV-16, 18, 52, 58, ect.) based on the risk of the virus to cause cancer in the uterine cervix [5].

It has been proved that long-term and persistent infection of HR-HPV is closely related to the occurrence and development of cervical cancer [6]. However, there are significant differences in HPV susceptible types in different regions. In China, HPV-16, HPV-52, and HPV-58 are the most common types, and the infection rate of HPV-58 is 15.31% [7], which is significantly higher than the global infection rate (4.7%) [8]. HPV-58 belongs to the Alpha-papillomavirus 9 (α9 HPV), and is relatively prevalent in China and other Asian countries [9]. HPV-58 genome can be divided into early region (E1, E2, E4, E5, E6, and E7), late region (L1 and L2) and long control region (LCR). Two HPV proteins, E6 and E7 (encoded by E6 gene and E7 gene, respectively), are the main drivers of cervical cancer development. The E6 protein binds to a ubiquitin ligase (E6AP) in the cell to form a complex, then binds specifically to p53 tumor suppressor protein. As a result, p53 is quickly degraded by proteases, resulting in decreasing the content of P53. E7 protein interacts with the retinoblastoma gene product (pRb), to inhibit the activity of the tumor suppressor protein through ubiquitination [10]. When HPV integrates into the host cell, E6 and E7 are invariably reserved and uncontrolled expressed, leading to the cell immortalization and malignant transformation [11–13].

Virus variations may cause differences in immune response and oncogenic potential [14]. There are some indications that the HPV-58 prototype and HPV-58 variants show a difference in their biological and biochemical characteristics to some extent. Several variations of HPV-58 have been reported in Latin America, Japan, southwest and northwest China [15–18], but few data were available on the variants of HPV-58 E6 and E7 genes in central China. In this study, we aim to analyze the gene polymorphisms and epidemic characteristics of HPV-58 E6 and E7 genes in Jingzhou area of central China and further evaluate the possible influence of E6-E7 sequence variations on immune reaction of antigens through describing the multiple putative B cell epitopes affected by the amino acid changes in the HPV-58 E6-E7. Our results could provide experimental data for the further study of therapeutic vaccines development, epidemiology, and prevention on HPV-58.

## Materials and methods

### Specimen collection

In this study, samples of cervical exfoliated cells were collected from the female patients with possible HR-HPV infection who underwent routine cervical screenings at the Department of Gynecology of Jingzhou Hospital from Spe. 2019 to Oct. 2021. All the samples were stored at 4 °C, and DNA was extracted within 1 day. Informed consent of patients had been obtained, and the study subjects’ privacy was protected before sample collection.

### HPV DNA extraction and typing

Based on the method of magnetic beads, DNA was extracted according to the instruction of the nucleic acid extracting kit (Guangzhou Magen Biotechnology Co., Ltd.). 4 μL extraction products were used for HPV typing as a PCR template. According to the instruction of the HR-HPV typing test kit (Shanghai ZJ Bio-Tech Co., Ltd.), DNA extraction products were detected through the method of real-time quantitative PCR, including 15 types of HR-HPV (16,18,31,33,35,39,45,51,52,56,58,59,66,68,82), with advantages of excellent sensitivity, specificity and typing accurately (minimum detection limit: 10^4^copies/mL; coincidence rate of positive reference: 100%; coincidence rate of negative reference: 100%; no cross-reaction with HPV-11, -6, -42, -43, -44, -73, -26, -53 types, Chlamydia trachomatis, Ureaplasma urealyticum, Neisseria gonorrhoeae, Mycoplasma hominis, Mycoplasma genitalium, Candida albicans, HIV-1, group B streptococcus). Finally, DNA extraction products of single positive samples of HPV-58 were selected and stored at − 80 °C for the follow-up experiments.

### PCR amplification and sequencing

The E6 and E7 genes were amplified from HPV-58 single positive samples. According to the reference sequence provided by GenBank (D90400), software Primer Primer 6.0 was used to design sequence-specific primers of HPV-58 E6 and E7 genes following primer design principles. All primers were synthesized by Sangon Biotech (Sangon Biotech (Shanghai) Co., Ltd.). As followed, E6 F:5′- TTGGGTCACATTGTTCATG-3′, E6 R:5′-TCATAGCAGAATAGGTCAGT-3′; E7 F:5′- ATTTCGGGTCGTTGGACAGG-3′, E7 R:5′- TCTGTACCACTATCGTCTGCTGTT-3′. 25 μL reaction volume included 2 μL HPV DNA extraction, 15.875 μL ddH_2_O, 2.5 μL 10 × Buffer (Mg^2+^ plus) (Takara), 2 μL dNTPs (Takara), 0.125 μL Taq polymerase (Takara), and 1.25 μL forward and reverse primers (20 μM) (Sangon Biotech). The amplification conditions of E6 were initial denaturation at 95 °C for 3 min, followed by 35 cycles of 94 °C for 45 s, 58.2 °C for 45 s, 72 °C for 1 min, and the 72 °C for 10 min. The E7 amplification conditions were initial denaturation at 95 °C for 3 min, followed by 35 cycles of 94 °C for 45 s, 62.1 °C for 45 s, 72 °C for 1 min, and the 72 °C for 10 min. The PCR products were analyzed by electrophoresis in 2.5% agarose gel stained with GelRed nucleic acid dye (Biotium), and then sent to Sangon Biotech for sequencing.

### Analysis of E6 and E7 genes

The nucleotide variants and amino acid substitutions were analyzed by comparing all sequencing results with HPV-58 reference sequence (D90400) via the DNAstar and NCBI blast.

### Phylogenetic analysis

The Neighbor-Joining tree of HPV-58 E6 and E7 sequences was constructed by MEGA 11 software, Kimura 2-parameter model was used, and the Bootstrap replications was set at 1000. The following reference sequences collected from GeneBank sequences database were used to construct the phylogenetic branches: D90400 (A1), HQ537752 (A2), HQ537758 (A3), HQ537762 (B1), HQ537764 (B2), HQ537774 (C), HQ537768 (D1), HQ537770 (D2) [19].

### Secondary structure analysis and selective pressure analysis

The online server PsiPred (http://bioinf.cs.ucl.ac.uk/psipred/) was used to predict the secondary structure of E6 and E7 amino acid sequences. The selective pressure was analyzed by PAML X software. The non-synonymous and synonymous nucleotide divergence (dN/dS) was calculated by Codeml program. Using the Bayes Empirical Bayes (BEB) analysis, the sites with the posterior probability ≥ 0.95 were identified as the positively selected sites.

### B cell epitope prediction

We predicted linear B cell epitopes of E6 and E7 proteins by online software ABCpred (ABCpred submission page (iiitd.edu.in)) according to the default parameters. The higher the predicted score, the better the affinity of the epitope.

## Results

### Epidemic characteristics of HR-HPV in Jingzhou, Hubei province

**Fig. 1:**
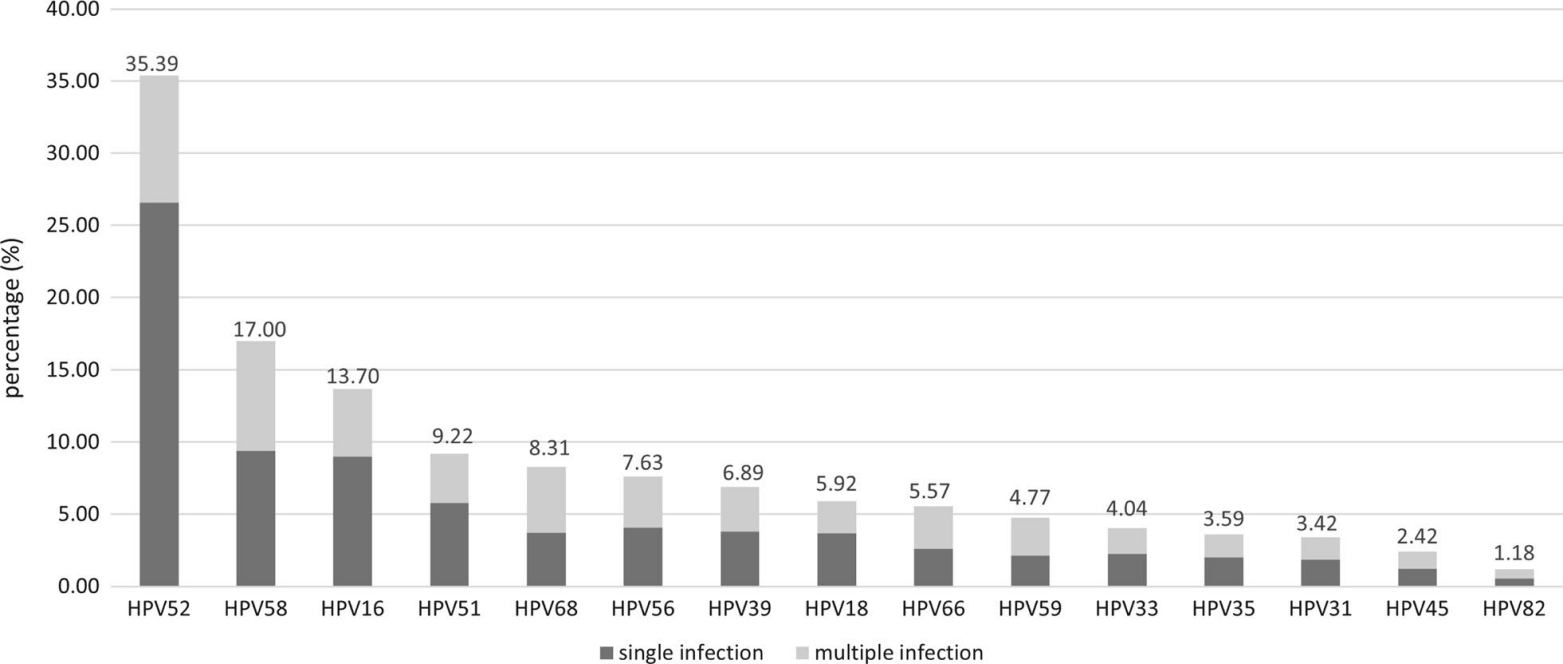
Prevalence of HPV in Jingzhou area. The horizontal axis shows different HPV types, the vertical axis shows infection rate. The numeric value above the histogram represents the HPV infection rate

A total of 18,266 samples were collected in Jingzhou Hospital, Yangtze University from Sep. 2019 to Oct. 2021. The overall prevalence of HR-HPV infection was 18.58% (3394/18266), with 14.62% (2671/18266) of participants being positive for a single HPV type, and the age group with highest infection rate was 58 to 67 years (32.43%). The detailed results are shown in Table 1. The most frequently detected HR-HPV types were type 52 (1201/3394, 35.39%), 58 (577/3394, 17.00%), 16 (465/3394, 13.70%) and 51 (313/3394, 9.22%), the results are shown in Fig. 1. In 577 HPV-58 positive samples, 319 cases (319/577, 55.29%) were single infection, 258 cases (258/577, 44.71%) were multiple infection. Among the HPV-58 co-infection, mixed infection with HPV-68 was the most common (77/258, 29.84%), followed by HPV-52 (76/258, 29.46%). The peak age of HPV-58 infection mainly at older than 67 years old and 57 to 67 years old, with the positive rate of 8.93% and 8.68%, respectively (Table 1).

**Table 1:** Multiple infections and positive for high-risk HPV infection status in different age groups [(n, %)]

Age group	Number of patients	Positive infection	Single infection	Multiple infection	Positive of HPV-58
< 18	19	2 (10.53)	2 (10.53)	0 (0.00)	1 (5.26)
18–27	1347	261 (19.38)	182 (13.51)	79 (5.87)	46 (3.41)
28–37	4826	766 (15.87)	626 (12.97)	140 (2.90)	101 (2.09)
38–47	6218	1009 (16.23)	837 (13.46)	172 (2.77)	157 (2.52)
48–57	4676	991 (21.19)	787 (16.83)	204 (4.36)	169 (3.61)
58–67	956	310 (32.43)	202 (21.13)	108 (11.30)	83 (8.68)
> 67	224	55 (24.55)	35 (15.63)	20 (8.92)	20 (8.93)
Total number	19,266	3394	2671	723	577

### Variation of E6 and E7 genes

There were 319 single positive samples of HPV-58, and some samples were failed amplified due to the low viral load or degradation of viral nucleic acid. Finally, 172 entire E6-E7 genes were successfully amplified and further analyzed. Compared with the reference sequence D90400, the prototype accounted for 18.02% (31/172) of the total sequencing samples, and the remaining 141 sequences were divided into 21 different variant groups named 58HB01-58HB21, which were submitted to the GenBank database and got the accession numbers were OL989036 to OL989056.

A total of 22 single nucleotide polymorphisms (SNPs) were observed in E6-E7 sequences. In E6 sequences, 10 SNPs were found, including 7 synonymous variants and 3 non-synonymous variants. The prevalent variants were A388C (K93N) (81/172) and C307T (57/172). In E7 sequences, 12 SNPs were observed, including 3 synonymous variants and 9 non-synonymous variants. T744G (104/172), G761A (G63D) (51/172), G694A (G41R) (47/172) and T803C (V77A) (46/172) were common variants. G261A (R51K), A310G, A340G and T407G in E6 sequence and A597G in E7 sequence were novel variants. Two non-synonymous variants (R51K and L100V) occurred in E6 sequences encoding the alpha helix, two non-synonymous variants (R9K and V77A) were found in E7 sequences encoding the strand. No insertion or deletion was detected in any sequence. The detailed results are shown in Table 2.

**Table 2:** The variations of HPV-58 E6-E7 gene

	E6										E7												
Positions	1	2	2	2	2	3	3	3	3	4	5	5	6	6	7	7	7	7	7	7	7	8	N
	5	1	2	5	6	0	1	4	8	0	9	9	3	9	2	4	5	6	6	6	9	0	
	2	7	9	9	1	7	0	0	8	7	7	9	2	4	6	4	5	0	1	3	3	3	
Reference	T	T	T	A	G	C	A	A	A	T	A	G	C	G	T	T	C	G	G	A	A	T	
58HB01	–	–	–	–	–	–	–	–	C	–	–	–	–	–	–	G	–	–	–	–	–	C	31
58HB02	–	–	–	–	–	–	–	–	C	–	–	–	–	–	–	–	–	–	–	–	–	–	31
58HB03	–	–	–	–	A	–	–	–	C	–	–	–	–	–	–	–	–	–	–	–	–	C	1
58HB04	–	–	–	–	–	T	–	–	–	–	–	–	–	A	–	G	–	–	A	–	–	–	43
58HB05	–	–	C	–	–	–	–	–	C	–	–	–	–	–	–	G	–	–	A	–	–	–	1
58HB06	–	–	–	–	–	T	–	–	–	–	–	–	–	–	–	G	–	A	–	–	–	–	1
58HB07	–	–	–	–	–	–	–	–	–	–	–	–	–	–	–	G	–	–	–	–	–	C	2
58HB08	–	–	–	–	–	–	–	G	C	–	–	–	–	–	–	G	–	–	–	–	–	C	1
58HB09	C	–	–	–	–	T	–	–	–	–	–	–	–	A	–	G	–	–	A	–	–	–	2
58HB10	–	–	–	–	–	–	–	–	C	–	–	–	–	–	–	G	–	–	A	–	–	–	1
58HB11	–	–	–	–	–	–	–	–	C	G	–	–	–	–	–	G	–	–	A	–	–	–	1
58HB12	–	–	–	G	–	–	–	–	–	–	–	–	–	–	–	G	–	–	–	–	–	C	1
58HB13	–	–	–	–	–	–	–	–	C	–	–	A	–	–	–	G	–	–	–	–	–	C	5
58HB14	–	–	–	–	–	–	–	–	C	–	G	–	–	–	–	–	–	–	–	–	–	–	1
58HB15	–	–	–	–	–	–	–	–	C	–	–	–	–	–	–	–	–	–	A	–	–	–	3
58HB16	–	–	–	–	–	T	–	–	–	–	–	–	T	–	–	G	–	A	–	–	–	–	9
58HB17	–	–	–	–	–	–	–	–	C	–	–	–	–	–	–	G	–	–	–	–	G	C	1
58HB18	–	–	–	–	–	T	–	–	–	–	–	–	–	A	–	G	A	–	A	G	–	–	2
58HB19	–	–	–	–	–	–	–	–	–	–	–	–	–	–	C	–	–	–	–	–	–	–	1
58HB20	–	–	–	–	–	–	G	–	C	–	–	–	–	–	–	G	–	–	–	–	–	C	1
58HB21	–	A	–	–	–	–	–	–	C	–	–	–	–	–	–	G	–	–	–	–	–	C	2
Reference AA	L	T	S	L	R	C	L	R	K	L	L	R	T	G	A	T	T	G	G	T	T	V	
AA Position	1	3	4	5	5	6	6	7	9	1	8	9	2	4	5	5	6	6	6	6	7	7	
	5	6	0	0	1	6	7	7	3	0			0	1	1	7	1	3	3	4	4	7	
										0													
AA Variant	–	–	–	–	K	–	–	–	N	V	–	K	I	R	–	–	N	S	D	A	A	A	
Secondary structure	H		H		S	H	H	H		S	H	H				S						H	

The nucleotides matching the reference (GenBank: D90400) are marked with a dash (–), AA, amino acid; S, strand; H, helix

### Phylogenetic tree

**Fig. 2:**
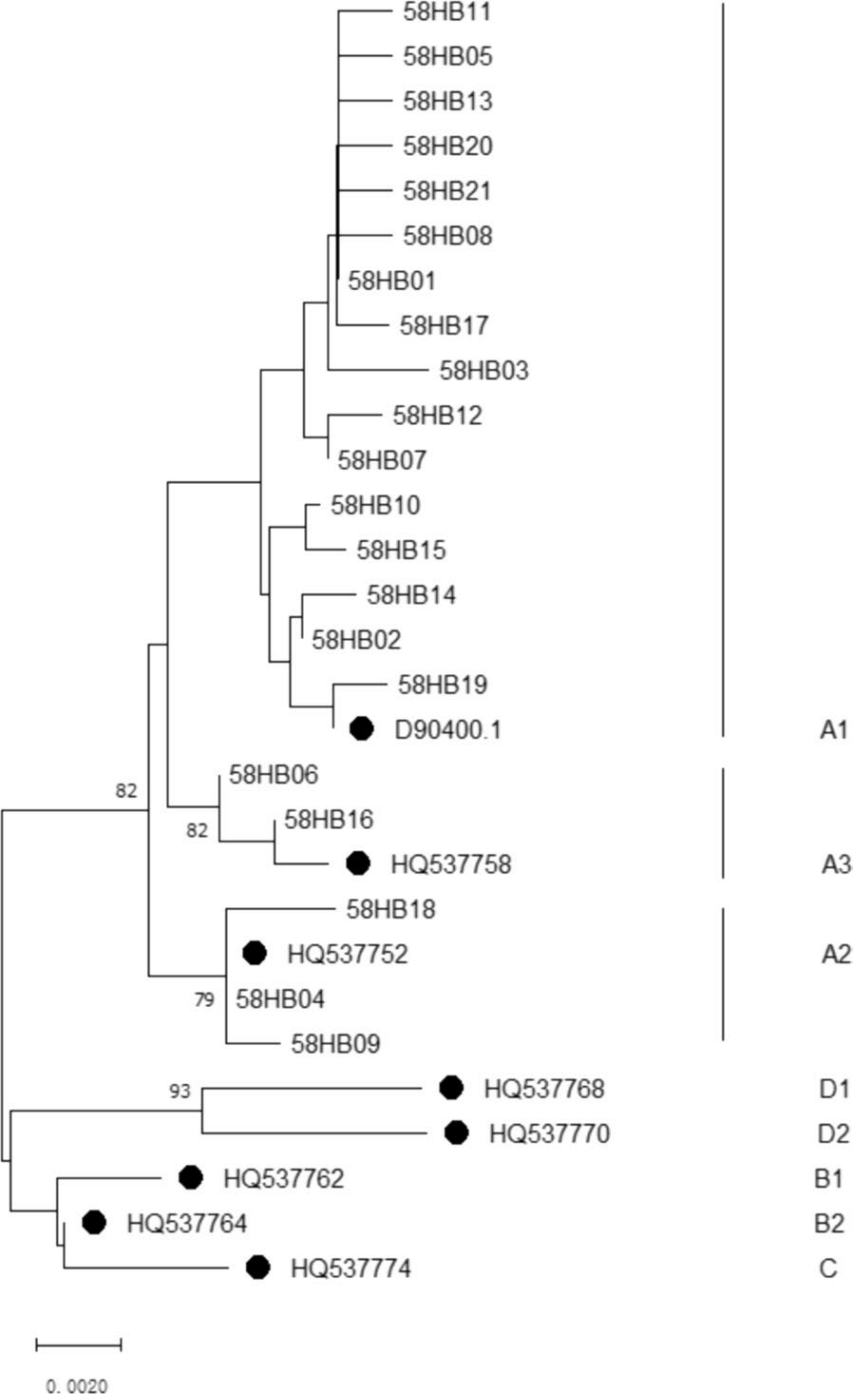
Neighbor-joining phylogenetic tree of HPV-58 E6–E7. The strains with black dot represented the reference sequences of sub-lineages, and the others were variant sequences. Only the bootstrap value > 70% was displayed

The phylogenetic tree was constructed with 21 unique HPV-58 E6-E7 sequences and 8 sub-lineage reference sequences by MEGA 11 software. The phylogenetic tree is shown in Fig. 2. All the variants were distributed in A lineage, with A1 sub-lineage was most abundant (115/172, 66.86%), followed by A2 (47/172, 27.33%) and A3 (10/172, 5.81%).

### Selective pressure analysis

The variable dN/dS rate ratios were tested among the various lineages using the PAML X. In HPV-58 E6 sequence, there was no positively selected site. In E7, codon site G63 was identified as the positively selected site. All the results are shown in Table 3.

**Table 3:** Positively selected sites of HPV-58 E6-E7 gene

Model	InL	Estimates of parameters	2∆InL	Positively selected sites
M7	− 1359.010172	p = 0.00500, q = 0.01173		NA
M8	− 1349.990137	P0 = 0.95895, p = 0.00500, q = 0.02017	18.04	G63**
		(p1 = 0.04105), ω = 8.65122	*P* < 0.05	

InL, log-likelihood difference between two models; 2∆InL, twice the log-likelihood difference between the two model; NA, not allowed; **, two asterisks mean indicate posterior probability ≥ 0.99

### B cell epitope prediction of HPV-58 E6 and E7 proteins

To evaluate the influence of HPV-58 E6 and E7 sequence polymorphisms on the host immune response and recognition, online server ABCpred was used to predict the potential B cell epitopes of E6 and E7 proteins. The epitope prediction scores should be greater than 0.85. The results are shown in Table 4. The occurrence of non-synonymous variants affected the B cell epitopes. For E6, the A to C substitution at the nucleotide site 388 led to the change of the amino acid site 93 (K to N), where B cell epitope YSLYGDTLEQTLKKCL (E6 81–96) was located, and the epitope scores decreased from 0.90 to 0.88. The T407G (L100V) variant located in the B cell epitope TLEQTLKKCLNEIL (E6 87–100), made the epitope scores decrease to 0.86. For E7, the non-synonymous variants R9K affected the B cell epitope RGNNPTLREYILDLHPEP (E7 2–19). G41R affected the B cell epitope DEDEIGLDGPDGQAQP (E7 33–48). T61N/G63D/T64A affected the B cell epitope YYIVTCCYCGTTV (E7 53–66), and G63S also affected this epitope. T74A and V77A affected the B cell epitope RLCINSTTTDVRLCINS (E7 67–82). Except G41R variant, which made the epitope prediction scores increase from 0.86 to 0.91, the scores of all the other variants dropped below 0.85.

**Table 4:** The putative B cell epitopes of HPV-58 E6 and E7 proteins

E6				E7			
Rank	Sequence	Start position	Score	Rank	Sequence	Start position	Score
1	YNYSLYGDTLEQTLKKCLNE	79	0.94	1	NYYIVTCCYTCGTTVRLCIN	52	0.92
2	PQEKKRHVDLNKRF	112	0.93	2	EIGLDGPDGQAQPA	36	0.91
3	YSLYGDTLEQTLKKCL	81	0.90	3	TCCYTCGTTVRLCINS	57	0.89
4	TGRCAVCWRPRRRQTQ	133	0.89	3	IGLDGPDGQAQPATANYY	37	0.89
4	TLEQTLKKCLNEIL	87	0.89	4	RLCINSTTTDVRTLQQ	67	0.88
5	KISEYRHYNYSLYG	72	0.87	5	DEDEIGLDGPDGQAQP	33	0.86
6	RCIICQRPLCPQEKKRHV	102	0.86	5	YYIVTCCYTCGTTV	53	0.86
7	GRWTGRCAVCWRPRRRQYQV	130	0.85	5	RGNNPTLREYILDLHPEP	2	0.86

## Discussion

Persistent infection of HR-HPV is the main factor for the occurrence and development of cervical cancer. The E6 and E7 proteins encode by E6 and E7 genes are the main carcinogenic proteins and play an important role in the cancer progression. HPV variants may be linked to the evolution of human groups to get an intrinsic geographical difference in prevalence and infections. The variants analysis of HPV E6 and E7 gene from clinical samples is of great significant for further study on vaccine and cervical cancer therapy in local area.

In this present study, we found that HPV-52, 58, and 16 types were prevalent types in Jingzhou area. The infection rate of HPV-58 was 17.00%, ranked second. This unusual high prevalence has also been reported in Chinese populations in Xi’an, Guangxi, and Chongqing [20–22].The prevalence of HPV-58 reached a peak when the patients were older than 67 years old in this study, suggesting that more attention should be paid to HPV screening in middle-aged women, which provided a basis for prevention, screening, and treatment of cervical cancer in Jingzhou area.

The results of E6-E7 sequencing showed that the sequence variability of E7 sequence (4.0%) was higher than that of E6 sequence (2.2%), suggesting that E6 may be a more suitable target for HPV therapeutic vaccine than E7. In E6 gene, C307T and A388C (K93N) were common variants. In E7 gene, T744G was most prevalent synonymous variant, G761A (G63D), G694A (G41R), and T803C (V77A) were common non-synonymous variants. There were four non-synonymous variants located in the sequences encoding the secondary structure of E6 and E7 proteins, may cause changes in oncoprotein folding and function, led to the differences in their ability to interact with tumor suppressor proteins, thus affecting the pathogenicity of HPV-58 [23]. Among the 21 variant groups, 58HB04, with the C307T/A694G/T744G/G761A variant, was most prevalent (43/172, 25.00%), which is consistent with a previous study in Colombian [24]. At present, there have been some researches on HPV-58 E6-E7 gene polymorphisms, and the common variants of E6 and E7 also have a high frequency in other areas [15, 16, 25–27]. Some studies have examined the HPV-58 E6 and E7 genetic diversity and their risk association with the development of cervical lesion. The A388C (K93N) substitution in E6 is more prevalent in women with normal cervix or low-grade lesions, shows a statistically significant negative trend of association with the severity of neoplasia in Hong Kong [28], which is consistent with the results of the study on HPV-58 in Shanghai area that A388C (K93N) substitution in E6 significantly reduces the risk of high grade squamous intraepithelial lesions (HSIL) [29]. In E7, the occurrence of C632T (T20I) and G760A (G63S) variants are positively correlated with the severity of neoplasia in Hong Kong [28], which is also found in a study in Zhejiang by Ding et al. [30]. The exact mechanism for the increased oncogenicity of the variants needs further investigation. G261A (R51K), A310G, A340G, T407G (L100V) of E6 gene and A597G of E7 gene were newly identified variants in this study, which have not been reported in previous studies, may provide real and effective data for the development of regional therapeutic vaccines. Our study can provide basic data for further studies of the relationship between E6-E7 gene variations and the severity of the cervix lesion.

HPV-58 type has four lineages of A, B, C and D. Lineage A is significantly more frequent in Asia than in America, lineage B is mainly distributed in America, and lineage C and D seem to be more frequent in Africa [31]. In this study, lineage A was most prevalent in Jingzhou area, especially for sub-lineage A1, which was also prevalent in Yunnan, Sichuan, Zhejiang etc. [16, 25, 32]. The higher oncogenicity and infection rate of sub-lineage A1 may explain the reason why HPV-58 has a high incidence of cervical cancer in China.

At present, all HPV vaccines on the market (Cervarix, Gardasil, and Gardasil-9) are prophylactic vaccines based on L1 protein immunogenicity, which play an effective role in preventing HPV infection by producing specific neutralizing antibodies. However, the vaccines do not prevent cancer development in individuals who are already infected [33]. Therefore, the development of therapeutic vaccines has broad application prospects in the management and control of HPV infection, and HPV E6 and E7 genes are ideal targets for the design of therapeutic vaccines against HPV [34, 35]. ABCpred server was used to predict the B cell epitopes of HPV-58 E6 and E7 proteins and analyzed the effect of amino acid changes caused by gene variation on the B cell epitope. In this study, only G694A (G41R) nucleotide substitution increase the epitope score of DEDEIGLDGPDGQAQP (E7 33–48), while the other variations all led to the decrease of epitope score. B cells play a significant role in HPV-related tumor immunotherapy and response to cervical lesion and cancers caused by HPV [36]. By analyzing the impacts of the occurrence of non-synonymous variants on the affinity of B cell epitopes, we can further understand their influence on the immune function of host cells. The B cell epitopes predicted in this study can be considered as potential candidates for therapeutic vaccines against HPV-58 development in the Chinese population, and the occurrence of amino acid substitution must be considered in the development of vaccine. These predicted epitopes need to be verified in vitro and in vivo experiments in subsequent studies.

This study analyzed the positive selection of HPV-58 E6 and E7 sequences in Jingzhou area. The main feature of positive selection is that it causes an unusual repaid increase in the frequency of alleles, which allows a species to adapt quickly to environmental changes [37]. The positively selected sites G63 found in E7 gene may have evolutionary significance in HPV-58 adaption, meaning that this variation is beneficial for the virus to adapt to the changes in the environment. However, relevant research is minimal, and more studies are needed to confirm this hypothesis.

## Conclusion

In this study, we analyzed the gene polymorphism of HPV-58 E6-E7 sequence and amino acid substitution of E6-E7 proteins in Jingzhou area, constructed a phylogenetic tree to analyze the lineage characteristics, and predicted the B cell epitope to clarify the regional specificity of HPV-58 gene variation. Some new variations and B cell epitopes were observed in this study, and we also got a general knowledge of the gene polymorphism of HPV-58 in Jingzhou area. This study can supplement the knowledge of gene diversity of HPV-58 in central China, and provide an experimental basis for subsequent studies on epidemiology, evolution, pathogenicity and therapeutic targets of HPV-58.

## Data Availability

The data generated during the current study are available in the NCBI repository (Home—Nucleotide—NCBI (nih.gov)). The sequence data were submitted to GenBank with accession numbers OL989036-OL989056.

## Abbreviations

HPV-58: Human papillomavirus type 58
HR-HPV: High-risk human papillomavirus
PCR: Polymerase chain reaction
SNPs: Single nucleotide polymorphisms
LCR: Long control region
HSIL: High grade squamous intraepithelial lesion
The amino acid acronyms R, K, N, L, V, T, I, G, S, D and A: Arginine, lysine, asparagine, leucine, valine, threonine, isoleucine, glycine, serine, aspartic acid, and alanine
